# A decade of publication trends in the Mountain West Clinical and Translational Research Infrastructure Network

**DOI:** 10.15761/jts.1000483

**Published:** 2025-09-05

**Authors:** Lorraine Evangelista, Reimund Serafica, Joseph Guerrero-Lopez, Dagmawit Teka, Xiangxue Xiao, Yingke Xu, Francisco S Sy

**Affiliations:** 1 Sue & Bill Gross School of Nursing, University of California Irvine, 854 Health Sciences Rd, Irvine, California 92617, United States; 2 School of Nursing and Mountain West CTR-IN, University of Nevada, Las Vegas, 4505 S. Maryland Parkway, Las Vegas, NV 89154, United States; 3 Mountain West CTR-IN, University of Nevada, Las Vegas, 4505 S. Maryland Parkway, Las Vegas, NV 89154, United States; 4 NW-SCORE, Department of Radiology, University of Washington School of Medicine, United States; 5 Mountain West CTR-IN and School of Public Health, Department of Environmental and Occupational Health, University of Nevada, Las Vegas, 4700 South Maryland Parkway, Las Vegas, NV 89154, United States

**Keywords:** translational research, scientometrics, grey literature, bibliometrics, health inequities

## Abstract

**Background:**

The Mountain West Clinical and Translational Research Infrastructure Network (MW-CTR-IN) promotes junior investigators’ careers *via* robust mentorship and faculty development. A key success indicator is the diversity and volume of scholarly publications. We employed scientometric methods to evaluate both database and non-database outputs—including academic works not traditionally indexed in bibliographic databases—over ten years, examining publication types, thematic focuses, and factors such as gender and discipline.

**Methods:**

We analyzed 1,554 peer-reviewed publications (1,141 from databases and 413 from non-database sources) published between 2014 and 2024. Publications were categorized by type and translational research stage using predefined criteria. Two independent coders classified the manuscripts. Gender and discipline differences were assessed.

**Results:**

From 2014 to 2024, 1,554 peer-reviewed publications were documented—1,141 database articles (68%) and 413 (31%) non-database outputs. Notably, publication numbers nearly doubled between 2020 and 2022 compared to previous years. Early database publications primarily focused on T0 and T1 levels (preclinical and basic research). In contrast, later years (2020, 2022, and 2023) saw significant growth in T3 and T4 research, indicating a shift toward advanced translational efforts. Males predominated in basic science (28% *vs*. 14%, p < .001).

**Conclusions:**

Rising publication trends underscore MW-CTR-IN’s success in building junior research capacity and reveal the remarkable impact of both traditional and non-traditional scholarly outputs in guiding equitable, interdisciplinary training. The inclusion of non-database publications—such as guidelines, protocols, and technical innovations—offers a more comprehensive evaluation of academic productivity and translational impact.

## Introduction

Translational research plays a crucial role in bridging the gap between scientific discoveries and meaningful health outcomes [1]. Despite their importance, we still lack a comprehensive understanding of how effective faculty development programs are, particularly in terms of their impact on academic production. Although previous studies have examined career advancement through grant acquisition and the quality of mentorship, there is a lack of quantitative evidence on how structured training initiatives impact publication performance and research diversity [2]. This gap is particularly significant in addressing health inequalities, as novel translational research is essential to develop targeted and effective programs for individuals lacking access to them [3].

The Mountain West Clinical and Translational Research Infrastructure Network (MW-CTR-IN) was established to assist junior researchers in overcoming these challenges by providing comprehensive assistance, mentorship, and training across several disciplines. The initiative aims not just to assist individuals in advancing their careers but also to promote research that directly tackles disparities in health outcomes among lower socioeconomic groups [4]. Nevertheless, to date, the project’s effectiveness has been predominantly assessed using conventional techniques, which creates a significant gap in our understanding of how various forms of scholarly output influence the evolving domain of translational science [5].

Prior assessments of Clinical Translational Service Award (CTSA) hubs and Institutional Development Award (IDeA)-state efforts have predominantly focused on database-indexed articles and conventional bibliometric criteria. They have overlooked the extensive array of non-traditional outputs that facilitate community engagement and implementation science. Yu, et al. [6] employed bibliometrics, social network analysis, and altmetrics to demonstrate the productivity and impact of NC TraCS; however, they omitted best-practice guidelines, study methodologies, and technical briefs from the scientific record. Kataoka-Yahiro, et al. [5] examined faculty development strategies and publication performance at Mountain West IDeA-state schools, focusing solely on indexed journal articles and grant outcomes [6]. They disregarded grey literature contributions, which frequently serve as crucial instruments for translating policy and practice. Our analysis addresses this critical gap by examining both database and non-database outcomes. It provides a more comprehensive overview of MW-CTR-IN’s academic influence.

This study aims to address two significant research inquiries: How does the publishing output demonstrate the effectiveness of the MW-CTR-IN faculty development program? What types of publications exist—database and non-database—and how do these outputs correspond to the various stages of translational research (T0–T4)?

Addressing these inquiries not only addresses a significant deficiency in the assessment of translational research training programs but also demonstrates their potential to enhance overall health equity. Enhanced dissemination of research findings, increased visibility for junior researchers’ contributions, and the incorporation of a broader spectrum of academic inputs can all facilitate the advancement of public health initiatives and the policy-making process [7].

To better contextualize the educational and institutional impact of MW-CTR-IN, this study also aligns conceptually with established frameworks for evaluating faculty development. For example, the Kirkpatrick Model prioritizes four types of training outcomes: reaction, learning, behavior, and results. It helps us understand how mentorship and support structures lead to actual scholarly outputs such as publications [8]. Another example of a multilevel structure is the Consolidated Framework for Implementation Research (CFIR), which examines how institutional settings, intervention characteristics, and individual factors interact to influence program performance [9]. Our primary research uses scientometric and translational categorization algorithms to examine trends in publications. However, these faculty development models demonstrate that publication productivity is a good indicator of larger changes in education and institutions [10].

Database-indexed publications have been the primary focus of evaluations of translational research programs throughout history. They have not taken into account non-database outputs, including technological advancements, research methodologies, and best-practice protocols. This omission is a result of the long-standing limitations of current bibliometric approaches, which primarily focus on indexed journal articles and lack mechanisms to include peer-reviewed grey literature. Consequently, significant contributions to implementation science and community-engaged research have not received adequate recognition in assessments of scholarly output. Our analysis addresses this deficiency by integrating results from both databases and non-databases, thereby providing a more comprehensive and equitable representation of academic impact. This approach aligns with current proposals to broaden research measurements, enhancing their relevance to translation and their contribution to society [11].

## Materials and methods

### Study design

We employed a scientometric methodology, a specialized subset of bibliometrics, to rigorously analyze the quantity, diversity, and thematic concentration of academic papers produced by researchers supported by MW-CTR-IN over a decade. This analysis is optimally conducted using scientometric approaches, since they provide quantitative data on publication trends that enable objective assessment of impact and facilitate the planning of future educational and policy activities.

### Study setting and participants

The MW-CTR-IN supports junior investigators at institutions such as the University of Nevada, Las Vegas. Since the first cohort completed projects in the fall of 2014, our analysis encompasses publications issued between 2014 and 2023. The program offers a range of resources, including mentorship and grant-writing training, to foster interdisciplinary research that directly addresses health disparities.

### Data collection

We obtained publications from both primary bibliographic databases (for database papers) and other sources that compile non-database publications. We identified database publications by systematic searches of PubMed, Scopus, and Web of Science utilizing the author’s name, the institution’s name, and the project’s identifiers. We verified publications against our grant report records to ensure comprehensive coverage.

We obtained non-database publications directly from the curriculum vitae (CVs) of researchers financed by MW-CTR-IN. These CVs were submitted annually as part of the program’s reporting requirements. The outputs encompassed elements typically absent from bibliographic databases, such as research methodologies, best practice guidelines, technological advancements, editorials, and commentaries. We verified all entries on journal websites or institutional repositories to ensure they were peer-reviewed and that the authors were consistent with the information provided.

We categorized annual publications into two classifications: database and non-database. We aggregated the data annually and created a dual-axis line graph including distinct trend lines for each publishing category. The x-axis represents calendar years, while the left and right y-axes denote counts and percentages, respectively. The discrepancies in the publication figures from previous revisions were resolved. The final compendium comprises 1,554 peer-reviewed publications, with 1,141 indexed in a database and 413 unindexed.

### Categorization, coding, and verification procedures

Publications were categorized by type (database *vs*. non-database) and assigned to a translational research stage (T0–T4) using an adapted version of the framework proposed by Fort, et al. [12] (Table 1).

**Figure 1.**
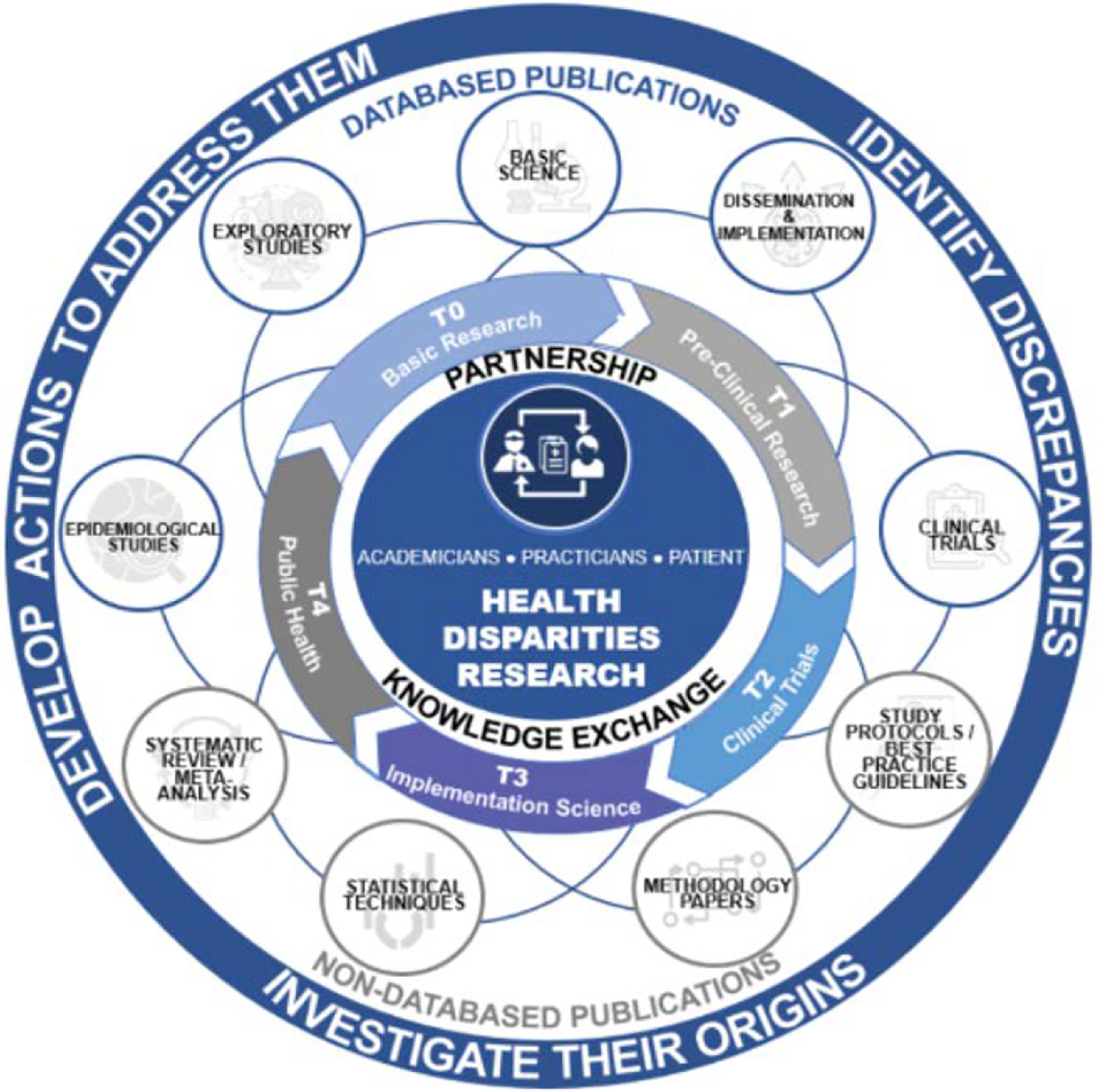
Translational research framework for classifying database publications (T0-T4)

Database articles in our study refer to peer-reviewed original research works that are indexed in major bibliographic databases. Figure 1 illustrates the categorization of these papers according to a translational research paradigm derived from Fort, et al. [12]. Following the parameters of this paradigm, publications are classified into the following stages. In the field of research, the term “basic research” (T0) refers to studies that explore the fundamental factors contributing to health and disease. These investigations can be conducted by animal trials, genome-wide association studies, preclinical research, and the analysis of extensive datasets. Proof-of-concept research, biomarker studies, therapeutic target identification, and preliminary drug development exemplify T1 applications that leverage initial findings in practical settings. T2 focuses on clinical research that directly impacts patient care. This includes formulating guidelines for evidence-based practice and helping with Phase I to IV clinical trials. T3 denotes implementation research, encompassing comparative effectiveness studies, pragmatic trials, health services research, and behavioral change programs. Finally, T4 pertains to population health research, which emphasizes epidemiology, policy, and environmental impact assessments, as well as prevention strategies and cost-effectiveness evaluations. Additionally, systematic reviews and meta-analyses synthesize existing evidence using systematic methodologies and statistical techniques and are included under this category [13–16].

Non-database articles encompass a broader range of scholarly outputs that are not typically indexed in traditional bibliographic databases. This category includes research protocols and best practice guidelines, where the former provides a comprehensive outline of a study’s objectives, methodology, and rationale [17], and the latter offers evidence-based recommendations to guide healthcare decision-making [18]. Moreover, non-database articles cover technical innovations—reports detailing novel techniques, modifications of existing methods, or new equipment—and an “others” category, which comprises case reports, commentaries, and editorials that offer expert insights, succinct overviews, or descriptive accounts of noteworthy clinical observations [16].

All non-database outputs were vetted to ensure they met the standards of peer review. Research protocols and best-practice guidelines were included only if journals or conference proceedings had accepted them following a formal external peer-review process. Technical innovation reports were limited to those published in refereed outlets (e.g., methods or engineering journals) or those that documented an external editorial review. Institution-only reports or internal white papers without evidence of external review were excluded. Where necessary, we confirmed peer-review status *via* journal websites, indexing platforms, or correspondence with the publishing editorial office.

Two independent coders reviewed and classified each manuscript based on title, abstract, and full text (when needed) [19,20]. The coders also extracted metadata related to the primary author’s gender and disciplinary background for subgroup analyses [21]. Discrepancies between coders were resolved through a consensus-based discussion. If disagreement persisted, a third reviewer adjudicated the final classification. Although inter-rater reliability metrics (e.g., Cohen’s kappa) were not formally calculated, disagreements occurred in fewer than 5% of publications, indicating high consistency in thematic and stage classification.

To quantify coding consistency, we conducted a pilot reliability assessment on a random 10% subset of publications (n = 155). A subsample of this size provides a representative basis for evaluating coder agreement while balancing feasibility constraints inherent in large-scale classification exercises. We generated the subset using the function in R (v4.4.3) to ensure unbiased selection. Both coders independently classified these items by publication type and translational stage, and we calculated Cohen’s κ for each dimension [22]. Agreement was substantial, with κ = 0.81 for database versus non-database categorization and κ = 0.79 for T0–T4 stage assignment. In the full dataset, discrepancies occurred in fewer than 5% of records; these were resolved through consensus discussion, with a third reviewer adjudicating persistent disagreements. This approach provides both a quantitative estimate of inter-rater reliability and reassurance that our dual-coding process yielded highly consistent classifications.

### Analytic approach and statistical framing

We employed the annual total of publications as the denominator for proportional comparisons to contextualize categorical distributions across the various stages of translational research (T0–T4). This method enables us to observe the evolution of publication focus and the temporal changes in research activity within the MW-CTR-IN. We employed coded content categories to identify theme trends and investigator-level data, such as gender and discipline, to compare publication forms and the emphasis on translation among subgroups.

### Statistical analysis

Statistical analyses were conducted using R (version 4.4.3, R Core Team 2024) [23]. To address the skewed distribution commonly found in bibliometric datasets, we employed descriptive statistics such as medians and interquartile ranges (IQRs) to define the publication outcomes [24]. The median number of publications per investigator was 4 (IQR: 2–7), indicating a right-skewed distribution characterized by a small cohort of highly productive authors.

We employed Mann–Whitney U tests to compare two independent groups (e.g., male versus female investigators) to identify any variations among investigator subgroups (e.g., gender, discipline). Kruskal–Wallis tests were applied when comparing three or more groups (e.g., across disciplinary clusters) [25]. To quantify the magnitude of subgroup differences, we calculated effect sizes for all inferential tests. For Mann–Whitney U comparisons (e.g., gender), we report the rank-biserial correlation (r). For Kruskal–Wallis H tests (e.g., discipline), we computed eta-squared (η^2^) using the formula η^2^ = H/(N – 1), where H is the test statistic and N is the total sample size. Dunn’s test was employed for pairwise comparisons post hoc, and r was calculated by dividing z by the square root of N.

For clarity, the main text had both percentages and absolute values (numerators and denominators). For instance, 1,141 (73.4%) of the 1,554 publications were indexed in a database, whereas 413 (26.6%) were not. A gender-based subgroup analysis revealed that male researchers authored 319 of the 1,141 database publications (27.9%), whereas female researchers authored 160 (14.0%), indicating a statistically significant difference (p < .001).

We employed the Cochran-Armitage test to analyze the temporal variations in the stages of translational research (T0–T4) [26]. This test examined whether the proportion of publications in later-stage research (T3/T4) significantly increased over time. The data indicated a statistically significant upward trend (χ^2^ = 18.7, df = 1, p < .001), confirming a transition from early-stage (T0/T1) to advanced translational outputs post-2020.

A significance level of 0.05 was utilized for all inferential comparisons, and effect sizes (such as r for the Mann–Whitney U test) were computed as necessary to contextualize the results. The effect size for the inequalities in publishing rates between men and women in fundamental research was r = 0.32, indicating a modest correlation.

## Results

**Figure 2.**
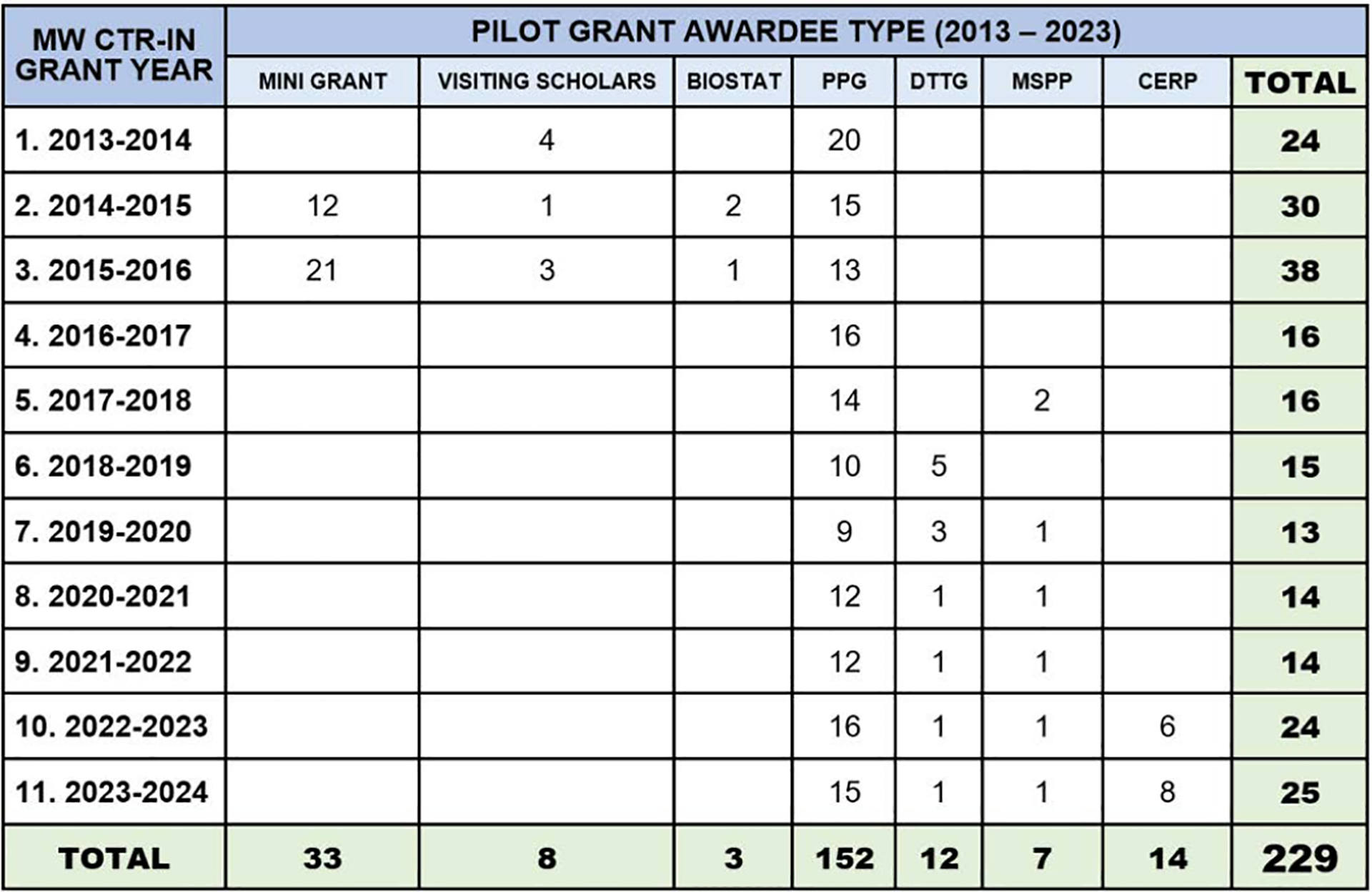
Annual distribution of database and non-database publications (2014–2024)

**Figure 3.**
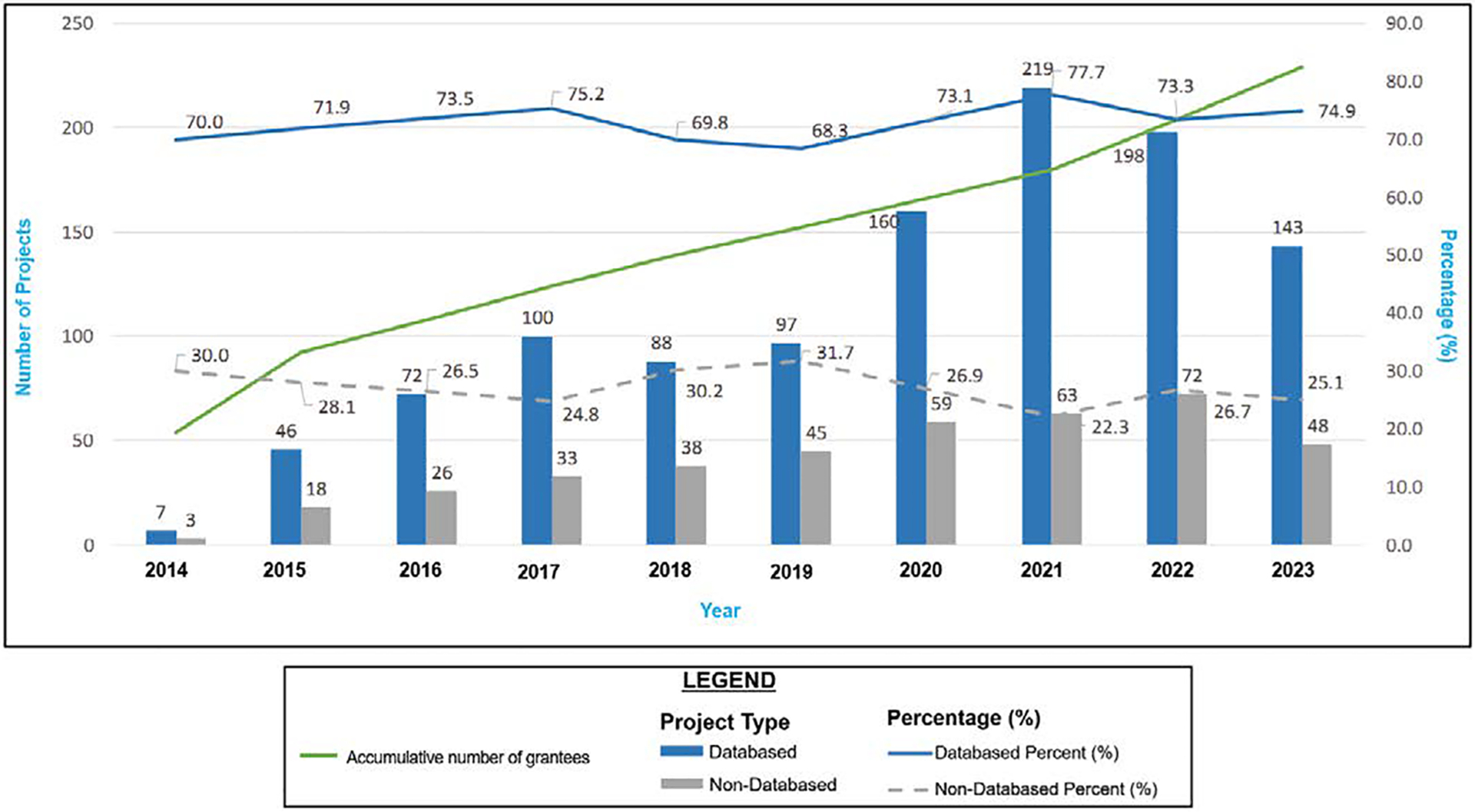
Temporal trends in total publications with pre- and post-pandemic comparison

**Figure 4.**
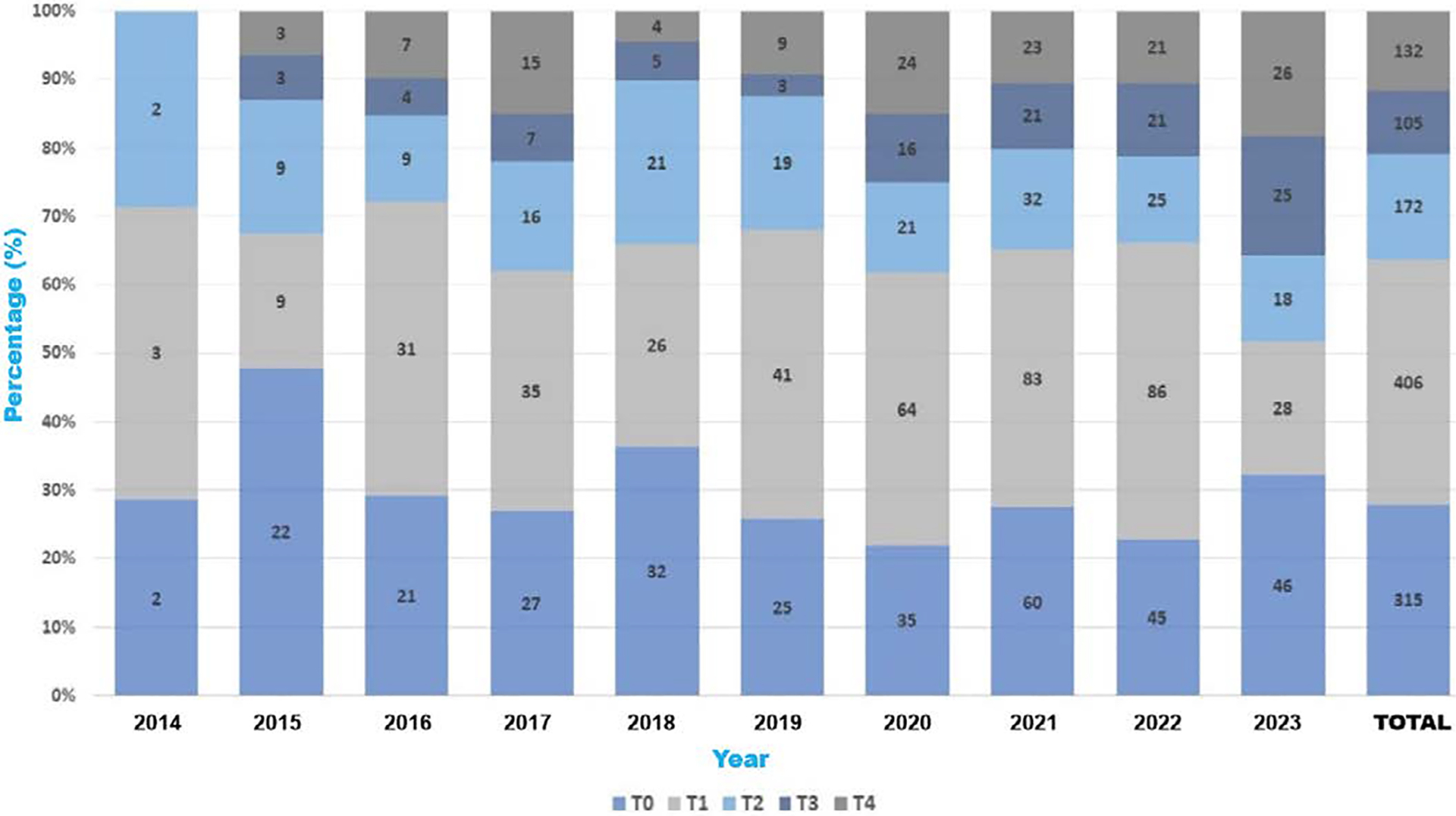
Distribution of database publications by translational research stage (T0-T4) from 2014 to 2023

Between 2014 and 2023, 1,554 peer-reviewed articles were published. Of these, 1,141 were database articles, whereas 413 were not. Over 68% of the annual outputs were derived from research, and more than 30% of the overall outputs each year consisted of non-database papers (Figure 2). (Figure 3) illustrates the temporal trend in publication volume, presented in percentages, showing a near doubling of total outputs from 2020 to 2022 compared to earlier years, followed by a slight decline in 2023 that remained approximately 1.5 times higher than pre-pandemic levels. A clear temporal trend is observed: The number of publications nearly doubled from 2020 to 2022, relative to the preceding years, albeit with a modest decline in 2023 that nonetheless maintained levels 1.5 times higher than those in pre-pandemic years (Figure 4).

During the 2020–2022 period, annual publication counts nearly doubled, from a pre-pandemic average of 110 papers per year (2014–2019) to 221 in 2022. This acceleration coincided with the widespread use of remote work practices and digital collaboration platforms, which accelerated manuscript preparation, review, and submission processes [27]. Concurrently, focused funding solicitations for COVID-19 and health equity research enabled investigators to redirect programs toward addressing timely public health issues, thereby streamlining the conventional grant-to-publication process [28]. Notably, non-database outputs, such as technical papers and best practice guidelines, increased correspondingly throughout this period, indicating a programmatic shift toward the rapid dissemination of translational tools and procedures tailored to meet emerging community needs [29].

We conducted a rapid internal content analysis of all 221 MW-CTR-IN articles from 2022 to substantiate the correlation between targeted COVID-19 financing, remote collaboration, and the increase in publications. We noted that 54 of these (24.4%) were unequivocally projects related to COVID-19 or SARS-CoV-2. Our findings represent a significant shift from the 2014–2019 timeframe, during which merely 4.7% of outputs were related to COVID-19 or SARS-CoV-2 research. The data also indicates that rewards and mentorship activities related to the pandemic have altered the themes. Likewise, internal findings mirror broader shifts in the research ecosystem: bioRxiv and medRxiv together registered a nearly 35% increase in COVID-19 preprint submissions during 2020 relative to 2019, reflecting the global adoption of rapid, open dissemination under conditions of remote work and accelerated peer-review pathways [30].

### Translational stage analysis:

Database publications primarily included studies at the T0 and T1 levels (preclinical and basic research) during the initial years. In contrast, the later years (2020, 2022, and 2023) saw significant growth in T3- and T4-level research. Figure 4 (properly labeled and credited) displays the distribution of publications across the T stages. A decade-long review of database publications reveals a marked transition from early-stage (T0/T1) investigations to later-stage (T3/T4) translational work [31]. Specifically, combined T3/T4 outputs increased from 0% (0/7) in 2014 to 35.7% (51/143) in 2023, with a notable jump from 12.4% in 2019 to 25.0% in 2020 as pandemic-related funding and remote workflows took effect. A year-by-year breakdown of T3 and T4 publications is presented in Table 2, illustrating the progressive rise in later-stage outputs from 2014 to 2023. A Cochran–Armitage test for trend confirmed this linear increase in advanced-stage publications over time (χ^2^_1_ = 18.7, p < .001). This shift mirrors MW-CTR-IN’s evolving mentorship priorities, which emphasize implementation science and population health, and align with the growing emphasis of funding agencies on health systems research [32]. Institutional evaluation metrics were also recalibrated to reward real-world impact, further encouraging junior investigators toward projects with immediate translational relevance [33].

### Thematic and disciplinary trends:

Publications encompassed topics such as non-communicable diseases, bioengineering, neuroscience, infectious diseases, and the social determinants of health. Notably, alongside biological investigations, several papers addressed emerging themes in environmental, psychological, and policy-related factors affecting health inequities.

### Gender and disciplinary differences:

Initial cohorts (2014–2015) had a predominance of male investigators (up to 90%); however, subsequent years demonstrated a more balanced gender representation. Statistical analysis revealed that males were significantly more engaged in basic science research (28% *vs*. 14%, p < .001), whereas females more frequently pursued public health research (12% *vs*. 5%, p < .001). Overall publication success rates did not differ by gender.

Gender-based analyses revealed that male investigators contributed 319 of 1,141 database publications (27.9%) versus 160 (14.0%) for females, a statistically significant difference (Mann–Whitney U = 45,230, p < .001) with a moderate effect size (r = 0.32). Disciplinary comparisons across three broad clusters (Basic Science, Clinical Research, and Public Health) were assessed using the Kruskal–Wallis H test, which yielded H(2) = 11.27, p =.004, η^2^ = 0.007, indicating a small effect. Dunn’s post-hoc pairwise tests showed a moderate impact between Basic Science and Public Health investigators (r = 0.30, p = .002) and a small effect between Basic Science and Clinical researchers (r = 0.18, p = .03); no significant difference emerged between Clinical and Public Health groups (r = 0.12, p = .15).

## Discussion

Publications remain a crucial element in advancing careers in health professions education as they disseminate innovative concepts, enhance professional visibility, and foster interdisciplinary collaboration [34]. Our examination of publications, both with and without databases, endorsed by the MW-CTR-IN, illustrates that systematic research training, mentorship, and support yield a varied range of excellent and notable academic outputs. The findings indicate that junior investigators from a range of disciplines and backgrounds have not only contributed rigorously to empirical research but also produced complementary works (e.g., systematic reviews, best practice guidelines, and technical innovations) that collectively enrich the translational research landscape.

This study’s translational research paradigm demonstrates that our junior researchers engage in a comprehensive spectrum, encompassing fundamental mechanistic investigations (T0), early-stage translational efforts (T1), clinical trials (T2), implementation (T3), and community health research (T4). The extensive spectrum demonstrates the commitment of MW-CTR-IN-supported scholars to fundamental scientific research and the application of their findings to enhance community and population health. Numerous studies emphasize the importance of basic research, highlighting the significance of understanding the cellular and molecular mechanisms that contribute to health disparities. Our findings indicate a shift in trend, with more funding allocated to research in the later stages (T3/T4) that supports both “bench-to-bedside” and “bedside-to-practice” initiatives. These are crucial methodologies for transforming laboratory findings into sustained health advancements [35].

This study contributes novel insights to the existing MW-CTR-IN literature by providing the first comprehensive and scientifically rigorous analysis of a decade’s worth of publications. This study differs from prior research that exclusively focused on programming components or singular case studies. It provides a comprehensive and long-term perspective on the research output across various phases of translation, genders, disciplines of study, and types of publication. This broader perspective provides supplementary insights into the program’s evolving scientific trajectory and the diversity of its researchers, thereby facilitating future strategic planning.

Our publication portfolio demonstrates that the integrated methodology of our program significantly enhances the dissemination of research findings and various modes of conveying academic work. This not only facilitates the advancement of junior researchers in their careers but also improves public health initiatives by guiding policy formulation and encouraging targeted actions [36]. The findings suggest that academic institutions and policymakers should invest in faculty development frameworks that encompass both conventional and unconventional scholarly outputs [37]. Our classification methodology includes a diverse array of research initiatives, including systematic reviews, technological innovations, and community-engaged implementation science. This establishes it as a paradigm that other IDeA states and research capacity-building efforts may adopt.

Beyond the conventional metrics of indexed journal articles, non-database outputs—such as technical reports, best-practice guidelines, research protocols, and commentaries—play a critical role in community engagement, policy translation, and health education. Co-developed standards and guidelines are “living documents” that local coalitions and community health workers can modify in real-time. This facilitates mutual learning between the two groups and fosters trust among individuals who lack access to healthcare. Technical briefings and editorial comments centered on methodologies frequently appear in practitioner workshops and continuing education courses, facilitating skill acquisition and discussions on novel translational tools. By equating these outputs with conventional publications, faculty development frameworks may more effectively demonstrate the whole spectrum of academic contributions that facilitate systemic change and health equity [38].

In parallel, the shift toward open-access models and preprint deposition has accelerated the dissemination of both database and non-database outputs, ensuring that findings reach practice and policy audiences without delay. Preprints enable investigators to share draft results publicly—often within days of submission—thereby inviting community feedback, fostering cross-institutional collaboration, and compressing the grant-to-publication lifecycle by several months in many cases [39]. Major funders and consortia now mandate or strongly encourage the posting of preprints as part of an “open science” initiative, recognizing that early, barrier-free access to evidence underpins rapid decision-making in public health emergencies and beyond [40]. Incorporating open-access and preprint training into MW-CTR-IN’s faculty development can therefore amplify the reach, relevance, and real-world impact of all scholarly outputs.

The MW-CTR-IN could enhance the use and accessibility of both database and non-database outputs by mandating open access and preprints. Aligning its faculty development requirements with the NIH’s 2025 Public Access Policy will ensure immediate availability of all NIH-funded works without complications. Simultaneously, including preprint deposition *via* bioRxiv, medRxiv, or specialized servers into program standards might significantly reduce the duration required to secure a grant and publish, potentially by weeks or even months [39]. It can solicit early feedback from the community and promote collaboration across institutions, utilizing remote and hybrid work methods. Systematic instruction on Creative Commons licensing, repository submissions, and adherence to journal regulations will equip researchers with the necessary tools to effectively utilize open-access avenues. These measures facilitate access to evidence for all, enhance its use for translation, and demonstrate MW-CTR-IN’s commitment to expeditious and equitable knowledge dissemination.

Our results indicate a significant shift towards T3/T4 research through 2023; nevertheless, it remains crucial to assess whether this emphasis on advanced phases will persist beyond the pandemic. Historical analyses of translational funding shifts—such as those following the 2008–2009 economic recession—suggest that without enduring mentorship priorities and aligned review criteria, early-stage (T0/T1) outputs can rebound as laboratory access normalizes [41]. To safeguard the momentum of implementation science at MW-CTR-IN, we recommend longitudinal tracking of year-on-year T-stage proportions and annual surveys of mentees’ research intentions. Grant review panels might also begin incentivizing proposals with explicit community-engagement or policy-translation milestones. By embedding these structural supports, the network can ensure that its pandemic-inspired gains in later-stage translational research persist well into the next decade.

Institutions seeking to evaluate or enhance their translational science pipelines may utilize this approach. The utilization of dual-coding, a specified T0–T4 stage classification, and the incorporation of both indexed and grey literature articles collectively contribute to a comprehensive documentation of a program’s scientific footprint [10]. This provides funding agencies and program managers a method to evaluate impacts, identify training deficiencies, and ensure that resources are aligned with high-yield translational objectives. If institutions implemented this methodology, it may facilitate program comparisons and aid in the formulation of more equitable and transparent policies in underfunded research areas [10].

As our results reveal, scaling translational research efforts by incorporating diverse disciplines and adopting comprehensive strategies to address health disparities remains a promising approach to reducing inequities. Moreover, integrating cutting-edge biomedical research with active community engagement is critical for achieving both scientific breakthroughs and their effective adoption by the populations that need those most [29].

### Strengths and limitations

A key strength of this manuscript is its robust methodological design, which employs a systematic categorization framework (Table 1) and adheres to established terminology. This study provides a comprehensive overview of scholarly production by systematically identifying publications across the translational research continuum (T0–T4). This enhances our comprehension of the progression of research [12]. The application of scientometric methodologies enhances objectivity through quantitative comparisons and trend analyses, facilitating the planning of future faculty development programs. The dual-coding method ensures consistent classification, thereby reducing bias and improving the reliability of thematic identification [42].

This study is distinctive in that it incorporates non-database outputs, such as research protocols, systematic reviews, technical innovations, and best-practice guidelines, in addition to traditional database-indexed articles. This provides a more comprehensive and equitable assessment of scholarly productivity and translational impact [43]. Indexed papers remain a primary metric for assessing research output; however, they frequently overlook peer-reviewed contributions that are crucial for disseminating information, engaging community members, and promoting policy initiatives. By integrating these diverse publication types, our approach moves beyond the conventional reliance on indexed articles as sole indicators of scholarly influence. It aligns with emerging calls for multidimensional evaluation frameworks that reflect quality, technological advancement, and varied dissemination pathways.

Although these methods have certain advantages, they also have some disadvantages that warrant consideration. Categorization is highly subjective because it is conducted in phases, particularly when determining the distinction between translational stages and identifying the type of publication that belongs in each category. Dual coding mitigates disparities, although classification schemes may vary between institutions. This indicates that forthcoming bibliometric analyses must be more uniform [44]. Furthermore, employing publication records as a proxy for research impact overlooks significant qualitative factors such as mentorship influence, interdisciplinary collaboration, and clinical implementation results. All of these factors are essential for a comprehensive assessment of faculty development programs [45]. Ultimately, while our scientometric review focused on outputs, subsequent research may employ models such as Kirkpatrick or CFIR to more thoroughly evaluate outcomes across the behavioral, institutional, and cultural dimensions of faculty development.

A further restriction pertains to the completeness and representativeness of the data. Although the study incorporates both indexed and non-indexed publications, variations in accessibility, reporting practices, and journal indexing may affect the total publication count [46]. Furthermore, gender and discipline-based analyses rely on available metadata, which may not fully capture the nuanced contributions of authors in multi-authored works [47].

Another important limitation pertains to author attribution in collaborative publications. Standard bibliographic metadata typically captures only author order and affiliation, without detailing individual contribution roles, making gender-disaggregated analyses vulnerable to undercounting the efforts of middle and supporting authors, who are more frequently women. This is especially pronounced in basic-science papers, where indexed outputs tend to highlight male senior authors and elevate their visibility over that of junior or female co-investigators [48]. By privileging first-and last-author positions in indexed databases, our approach may inadvertently bias findings toward male-authored basic research outputs, thereby obscuring the full breadth of collaborative contributions and skewing interpretations of gender equity in scholarly productivity [49].

A further drawback pertains to our reliance on English-language and English-published findings, which may introduce potential language and publication biases, thereby affecting representativeness. Most prominent bibliographic databases exclusively encompass English-language journals. This indicates a deficiency in the number of articles published in several languages, resulting in a “Tower of Babel” bias in systematic analysis. Excluding non-English trials may alter the outcomes of a meta-analysis [50]. This may render the outcomes of English-language research more favorable than they are [50]. Publication bias suggests that research yielding statistically significant or positive results is more likely to be submitted and accepted, resulting in the underpublication of null or negative outcomes. A thorough examination revealed that more than 50% of clinical studies yielding null outcomes remain unpublished, which suggests that our output metrics may overestimate program productivity and exclude valuable insights regarding efforts that had no significant effects [51].

Future studies should explore enhancements to scientometric methodologies, such as citation analysis and AI-driven network analysis, to better assess the efficacy of translational research [52]. Standardizing classification criteria throughout colleges and universities and incorporating qualitative assessments, such as investigator interviews or survey-based impact evaluations, could enhance our understanding of the effectiveness of faculty development initiatives [53].

## Conclusion

The MW-CTR-IN has significantly benefited junior researchers over the past decade. This has enhanced research productivity and facilitated collaboration among researchers across various domains of translational science. This project has substantially enhanced academic output through structured mentorship, organized faculty development, and meticulous scientometric analysis. This study illustrates the value of specialized training programs in preparing future translational researchers. These programs equip individuals with the necessary skills and resources to facilitate significant scientific discoveries and enhance healthcare outcomes.

The findings indicate progression of the program from basic research (T0) to advanced implementation (T3) and population health studies (T4). This suggests that an increasing number of individuals are striving to translate research findings into practical solutions for resource-limited settings. Various types of publications, including systematic reviews, best practice guidelines, and technological advancements, demonstrate the diverse methods for disseminating research findings. This ensures that data is utilized not only by researchers but also in clinical settings, as well as in public health programs and the legal systems. The utilization of AI-driven scientometric assessments and the promotion of improved collaboration among various disciplines can enhance the utility and fairness of research in future versions of faculty development programs, such as MW-CTR-IN.

This study highlights the significance of structured faculty development programs in fostering sustainable, high-impact translational research. MW-CTR-IN remains an effective mechanism for promoting quality research and advancing equitable healthcare solutions, as it continually adapts to emerging scientific findings and societal needs. Exploring study areas characterized by established gender disparities, with a particular emphasis on cross-disciplinary collaboration, is advisable to facilitate the equitable advancement of scientific knowledge.

## Figures and Tables

**Table 1.** Publication categories and translational stage definitions. It presents the definitions and examples of publications in categories within clinical and translational research, including both database and non-database examples. The T0-T4 table represents a progression of research phases aimed at translating basic scientific discoveries into practical applications that improve human health

Category	Definition	Examples/Notes
**Databased**	Publications presenting new primary data, including original research, Phase I–IV clinical trials, and reclassified systematic reviews/meta-analyses that address focused research questions with detailed methodologies.	Basic science, preclinical/animal studies, clinical research, systematic reviews.
**Non-Databased**	Articles (e.g., case reports, editorials, commentaries) that provide insights, best practice guidelines, or technical innovations but do not present new primary data.	Research protocols, technical innovations, and opinion pieces.
**Translational Stages (T0 – T4)**	Publications presenting new primary data, including original research, Phase I–IV clinical trials, and reclassified systematic reviews/meta-analyses that address focused research questions with detailed methodologies.	Categories adopted per Fort, et al. [12], and methodological recommendations by Mingers, et al. [42].

**Table 2.** Year-by-Year distribution of database publications by T3/T4 stage (2014–2023)

Year	Total DB Publications (n)	T3 Publications (n, %)	T4 Publications (n, %)	Combined T3 & T4 (n, %)
2014	7	0 (0.0%)	0 (0.0%)	0 (0.0%)
2015	46	3 (6.5%)	3 (6.5%)	6 (13.0%)
2016	72	4 (5.6%)	7 (9.7%)	11 (15.3%)
2017	100	7 (7.0%)	15 (15.0%)	22 (22.0%)
2018	88	5 (5.7%)	4 (4.5%)	9 (10.2%)
2019	97	3 (3.1%)	9 (9.3%)	12 (12.4%)
2020	160	16 (10.0%)	24 (15.0%)	40 (25.0%)
2021	219	21 (9.6%)	23 (10.5%)	44 (20.1%)
2022	198	21 (10.6%)	21 (10.6%)	42 (21.2%)
2023	143	25 (17.5%)	26 (18.2%)	51 (35.7%)

Note. Cochran–Armitage test for trend: χ^2^_1_ = 18.7, p < 0.001.
